# Montmorillonite immobilized Fe/Ni bimetallic prepared by dry in-situ hydrogen reduction for the degradation of 4-Chlorophenlo

**DOI:** 10.1038/s41598-019-49349-w

**Published:** 2019-09-16

**Authors:** Shuo-Shuo Zhang, Ning Yang, Xuming Zhuang, Liying Ren, Vinothkumar Natarajan, Zhaojie Cui, Hongyu Si, Xiaohan Xin, Shou-Qing Ni, Jinhua Zhan

**Affiliations:** 1 Shandong Provincial Key Laboratory of Soil Conservation and Environmental Protection & Shandong Provincial Key Laboratory of Water Pollution Control and Resource Reuse, Linyi & Jinan, PR China; 2 College of Chemistry and Chemical Engineering, Yantai University, Yantai, P.R. China; 3 Key Laboratory for Colloid & Interface Chemistry of Education Ministry, Department of Chemistry, Shandong University, Jinan, 250100 PR China; 4 Energy Institute, Qilu University of Technology (Shandong Academy of Sciences), Jinan, PR China; 5 Shandong Ztser Biological Technology Co., Ltd., Jinan, PR China

**Keywords:** Pollution remediation, Porous materials

## Abstract

This study puts forward a new way to produce montmorillonite immobilized bimetallic nickel-iron nanoparticles by dry *in-situ* hydrogen reduction method in the non-liquid environment, which effectively inhibits the oxidation of iron and nickel during the synthesis process and improves the reactivity of the material. The degradation of 4-Chlorophenol (4-CP) was investigated to examine the catalytic activity of the material. The morphology and crystal properties of the montmorillonite-templated Fe/Ni bimetallic particles were explored by using scanning electron microscopy, transmission electron microscopy, X-ray diffraction studies, and energy dispersive X-ray spectroscopy analysis. Results suggest that Fe and Ni particles were homogeneously dispersed on the montmorillonite. The optimization of Ni content and reduction temperature over the degradation of 4-CP was also studied. The introduction of Ni intensely improved the degradation of 4-CP and reached over 90% when Ni content was 28.5%. The degradation rate increased significantly with the increase of reduction temperature and showed maximum activity at the reduction tempreature of 800 °C. This study offers a new method to fabricate montmorillonite immobilized Fe/Ni bimetallic nanoparticles in the non-liquid environment and the composites exhibited high degradation activity to chlorinated organic compounds.

## Introduction

Chlorinated organic compounds (COCs) are one of the most important persistent compounds and have been detected in various environmental matrices. Chlorinated organic compounds have potential threats to both ecosystem and human health^1–5^. In addition, COCs and its derivatives have low biodegradability even at very low concentrations^6–9^. The nanoscale zero-valent metals is considered to be a promising strategy for the removal of COCs in contaminated environments^10–12^. Due to various factors such as low cost, abundance, non-toxic, excellent chemical, surface, and adsorptive properties, nZVI based materials are considered to play an important role in catalysis^13–18^. Further, in order to enhance the dechlorination efficiency, the deposition of secondary catalytic metal onto the surface of nZVI is necessary^19–21^. Several studies have demonstrated nZVI combined with second passive metals (e.g. Pd and Ni) resulted in effective dechlorination of PCBs, and the degradation efficiency could increase 3-fold compared to nZVI^22–27^. In a bimetallic system, the reductive metal (nZVI) plays the role of electron donor to dechlorinate the contaminants, whereas the secondary metal (for example Ni) accelerates the rate of dechlorination by preventing the conversion of chlorinated products into toxic end products. The corresponding mechanism of such reductive reactions was described that the second passive metals activate the hydrogen produced by the corrosion of nZVI, which subsequently attacks the aromatic ring and replaces –Cl through a hydrodechlorination process^25,28^. Researchers have found that nanoscale bimetallic catalyst composed of Ni and Fe exhibited much higher activity for the degradation of chlorinated organic compounds^19,29^. However, it cannot be ignored that nZVI catalysts have some disadvantages, such as its lack of stability, easy aggregation, easy passivation. The activity, stability and the surface area of nZVI decreased, when nZVI particles agglomerated into microscale particles due to its high surface energies and intrinsic magnetic interactions^15,30^. Therefore, prevention of aggregation and stability improvements of nZVI are major issues during the synthesis and application. To overcome this problem, several types of supporting materials have been investigated^17,31–34^.

Montmorillonite is an environmental friendly layered clay mineral with specific surface areas ranging from 700 to 800 m^2^g^−1^ ^35,36^. It is widely used as an absorbent for the toxic heavy metals and organic contaminants because of its high surface area, chemical stability, low cost and numerous structural properties^37,38^. He *et al*. loaded MnO_2_ nanosheets on montmorillonite by one-pot hydrothermal method and found that honeycomb-like MnO_2_ nanosheets vertically grew on the surface of montmorillonite^39^. MnO_2_ nanosheets@MMT exhibited high methylene blue removal rate^39^. In addition, many researchers have reported that montmorillonite showed the potential to prevent the agglomeration of ZVI^35,40,41^. Zhang *et al*. demonstrated that montmorillonite-templated Fe/Ni could selectively degrade more toxic PCB 36^42^. The degradation process was initiated by the intercalation of COCs into clay interlayers and subsequently reduced by Fe/Ni bimetal via a hydrodechlorination reaction^42^. Charles *et al*.^37^ explained that the adsorption of contaminants on montmorillonite surfaces was the key step for the dechlorination reactions. Also, researchers suggested that the exchangeable cations in clay interlayer played a dominant role in the adsorption of organic compounds from water^37^.

In general, montmorillonite-templated Fe/Ni bimetallic particles preparation process involved co-precipitation method and ion exchange/adsorption method, which occurs in the liquid phase^34,42^. In simple terms, ion exchange/adsorption method was prepared by the ion exchange between the metal cation and the surface cation of the supporting materials, or the metal cation was adsorbed on the surface of the supporting materials, and then the metal ion on the materials was reduced by the strong reducing agent to form the zero-valent particles of the bimetal directly on the supporting material^42,43^. However, during these syntheses processes, nZVI was easily oxidized with water, which resulted in reduced activity of Fe/Ni bimetallic particles. Thus, it is highly desirable to design an efficient preparation method to fabricate montmorillonite immobilized Fe/Ni bimetallic nanoparticles in the non-liquid environment.

In this work, montmorillonite-templated Fe/Ni bimetal was prepared in the non-liquid environment by a newfangled method for the dechlorination of 4-CP in aqueous solutions. By this method the ion exchanged montmorillonite was dry-reduced using hydrogen gas in a tube furnace. Thus, the oxidation of iron and nickel by water could be inhibited and their catalytic activity could be improved. 4-Chlorophenol (4-CP) was selected as a model pollutant to evaluate the dechlorination activity of Fe/Ni bimetallic particles. Transmission electron microscopy (TEM), scanning electron microscopy (SEM), X-ray diffraction (XRD) and energy dispersive X-ray spectroscopy were used to characterize the change in physicochemical properties of the Fe/Ni bimetallic system. Further, potential parameters which would affect the dechlorination efficiency of 4-chlorophenol by montmorillonite-templated Fe/Ni bimetal particles were also investigated.

## Results and Discussion

### Characterization of montmorillonite-templated Fe/Ni bimetal material prepared by dry ***in-situ*** hydrogen reduction

**Figure 1:**
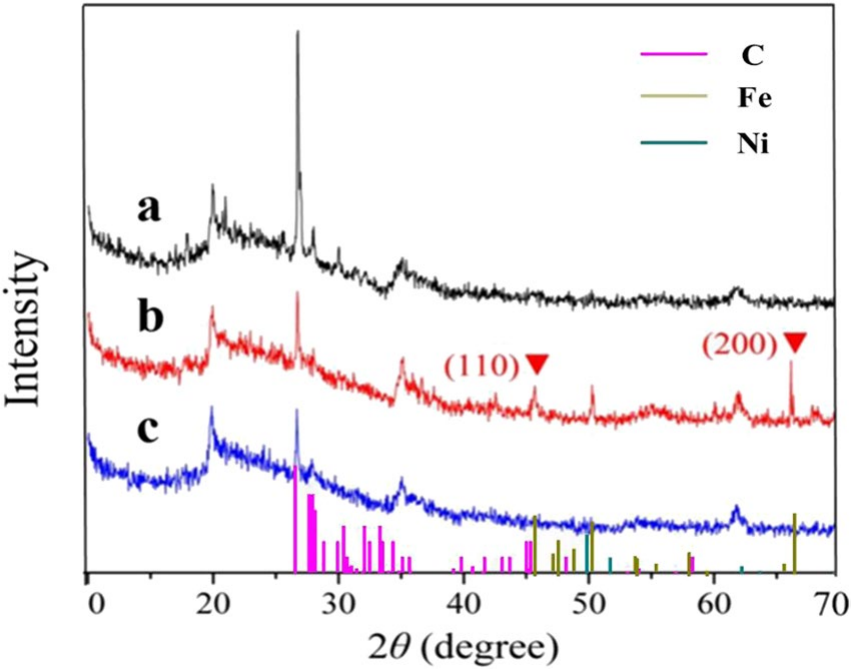
(**a**) X-ray diffraction spectra of montmorillonite, (**b**) Fe-montmorillonite and (**c**) Fe/Ni-montmorillonite.

**Figure 2:**
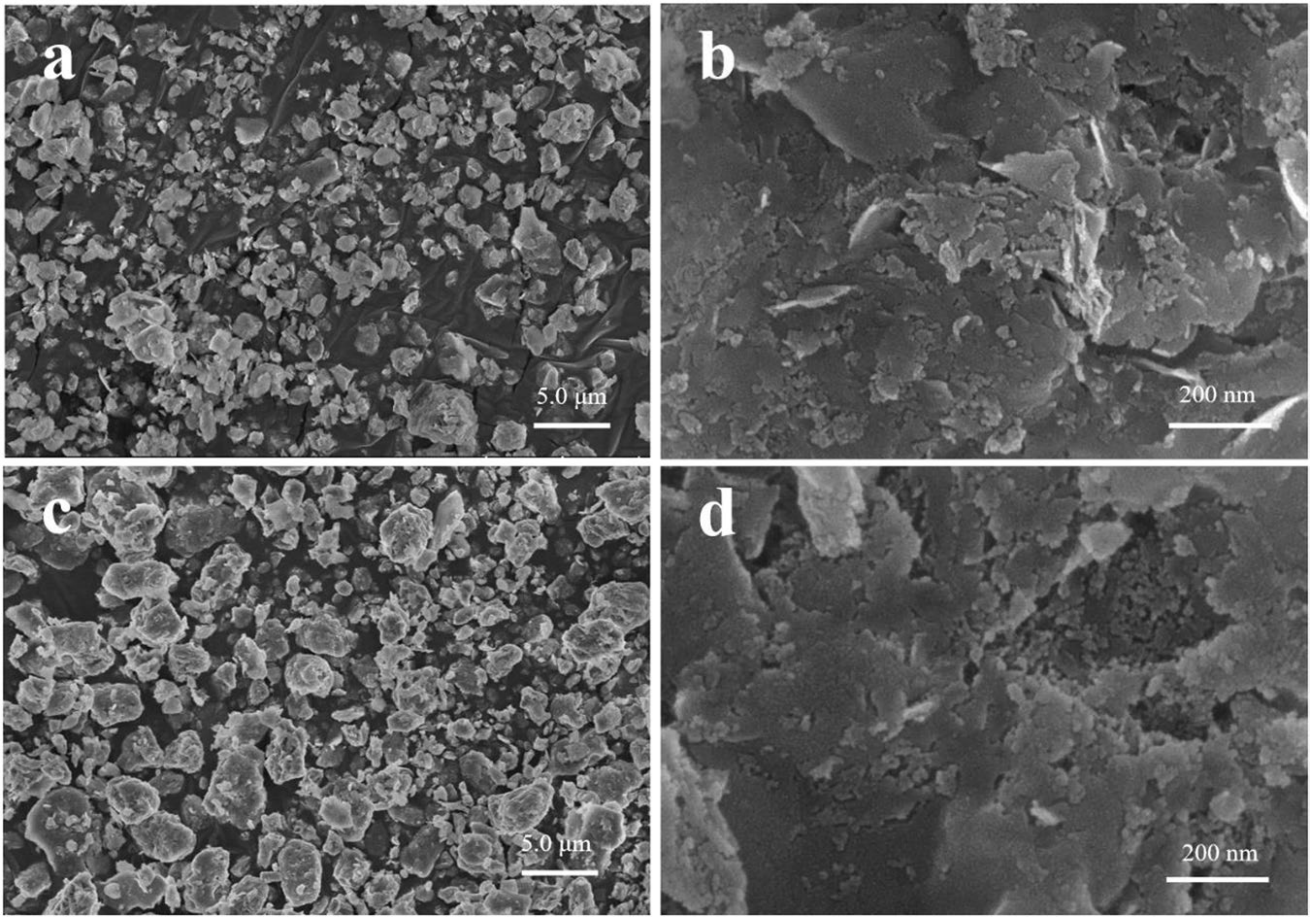
(**a**,**b**) SEM micrographs of montmorillonite and (**c**,**d**) Fe/Ni-montmorillonite.

XRD patterns of montmorillonite-templated Fe/Ni bimetal material were presented in Fig. 1. The characteristic diffraction peaks of montmorillonite at 19.89°, 26.64°, 35.30°, 62.80° were ascribed to the typical crystal structure of bentonite^44,45^. After montmorillonite modified with Fe and Ni, the intensity of the corresponding diffraction peaks were relatively weak, but the peak position didn’t change, which indicated the crystal structure of montmorillonite was not destroyed after loaded^46^. It can be seen that the Fe-montmorillonite exhibited diffraction peaks at 45.7° and 66.1°, suggesting the presence of iron in the crystal structure^19^. It is worth noting that the characteristic peak of Fe in Fe/Ni-montmorillonite rapidly decreased compared with the Fe-montmorillonite. Fe crystallites were smaller in size and more diffuse in Fe/Ni-montmorillonite could explain this phenomenon. In the diffraction pattern of the catalysts, no characteristic diffraction peak of Ni was observed, proving that Ni was highly dispersed on the surface of montmorillonite and may be close to amorphous or single-layer distribution. The above results showed that the addition of Ni could effectively promote the miniaturization of Fe grains and prevent the agglomeration of Fe^47^. The surface morphology of the different montmorillonite composites was shown in Fig. 2. It could be clearly seen that Fe/Ni particles were well dispersed on montmorillonite surface with no agglomeration. SEM images showed that the surface of montmorillonite exhibited irregular morphologies with a rough surface. After the addition of Fe/Ni bimetals, the roughness of montmorillonite became smaller, suggesting that Fe/Ni particles were successfully bonded to the surface of montmorillonite^46^.

**Figure 3:**
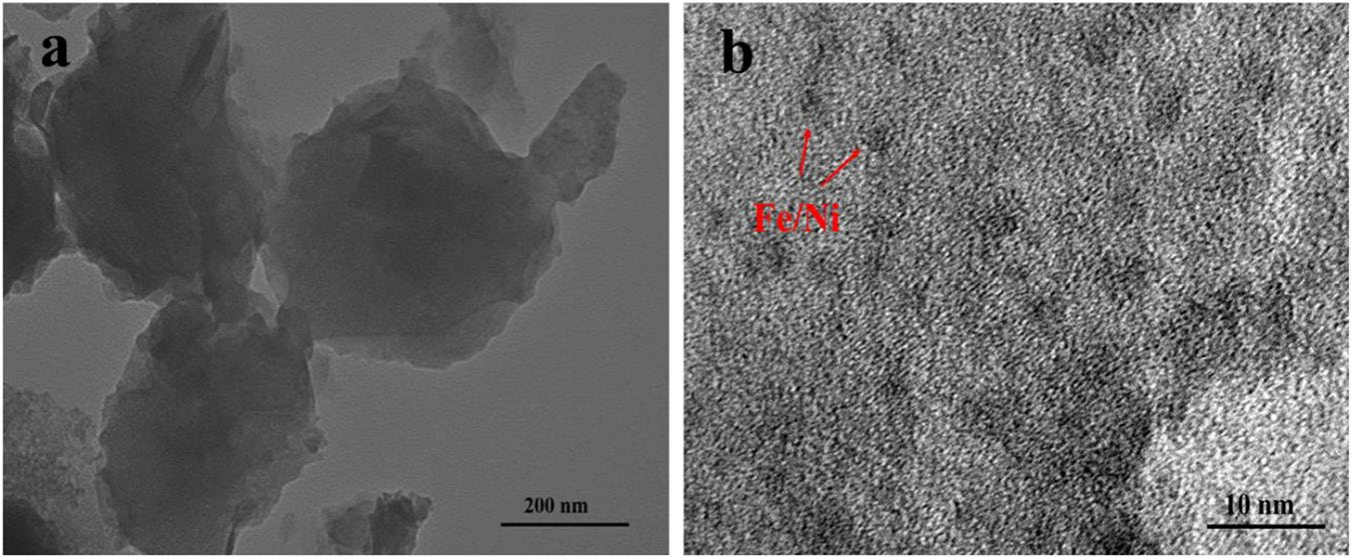
(**a**) Low-resolution TEM image and (**b**) high-resolution TEM image of Fe/Ni -montmorillonite material.

**Figure 4:**
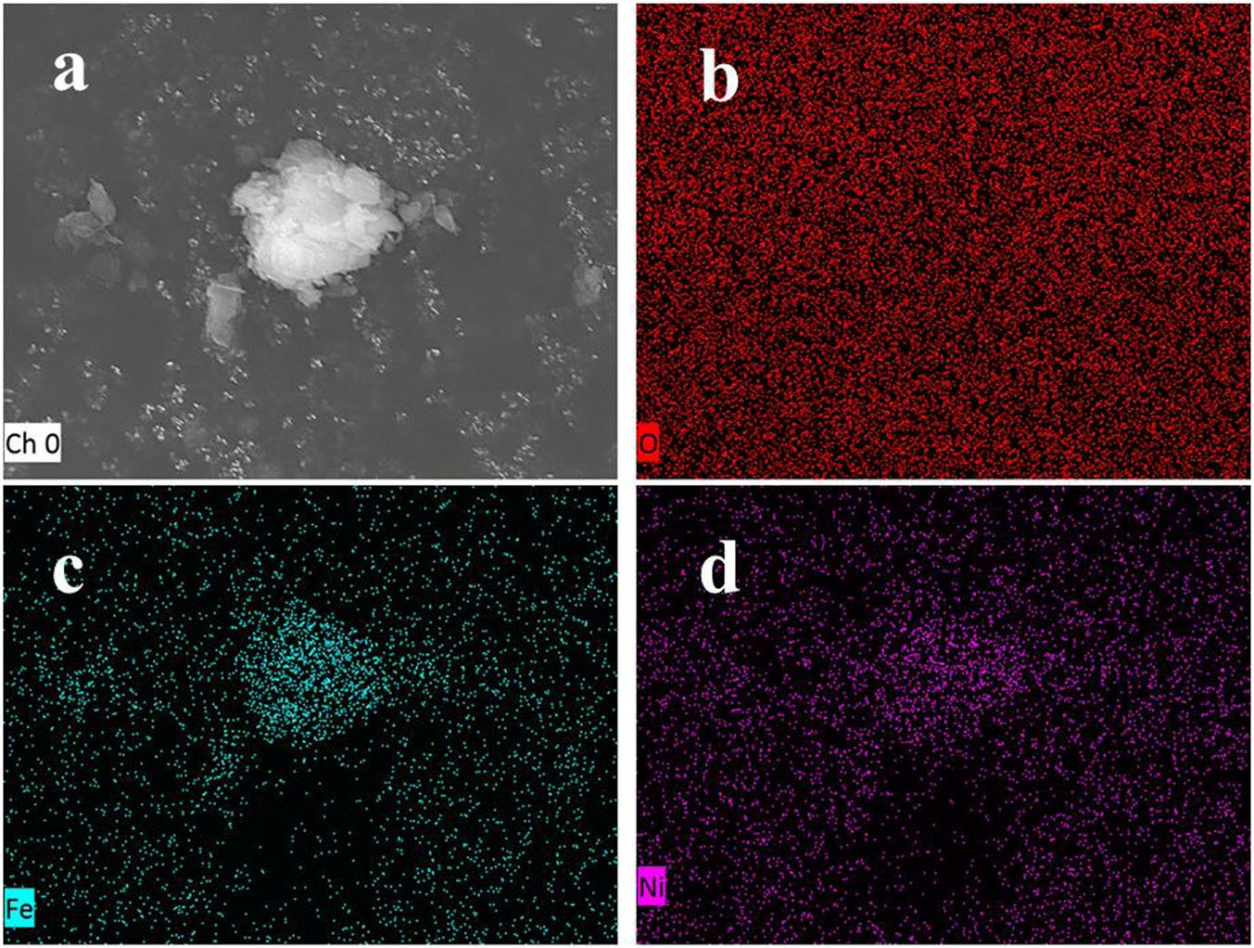
SEM images of (**a**) Fe/Ni -montmorillonite, EDS images of (**b**) O mapping, (**c**) Fe mapping and (**d**) Ni mapping of Fe/Ni montmorillonite material.

The morphology of Fe/Ni-montmorillonite (Fig. 3a) was found to be of spherical shapes (400–600 nm in diameter) which were connected in chains of beads probably due to the electronic and magnetic interactions between the metals^34^. The morphology of Fe/Ni-montmorillonite was further observed by high-resolution TEM and is shown in Fig. 3b. Based on the observation, Fe/Ni bimetallic particles of 3.0–5.0 nm size were well dispersed on montmorillonite. These results indicated that the novel method for montmorillonite immobilizing bimetallic Fe/Ni nanoparticles is highly effective. Figure 4 showed the EDS element mapping of Fe/Ni-montmorillonite particles synthesized by dry *in-situ* hydrogen reduction. The presence of Fe and Ni elements were confirmed from the EDS mapping images. Results obviously demonstrated that the bimetals were homogeneously distributed in the Fe/Ni montmorillonite material, and montmorillonite could effectively inhibit the agglomeration of Fe/Ni particles. Uniformly distribution of Ni and Fe made active sites exposed, which facilitated the contact of target contaminants with active sites and improved the degradation of the catalyst^47^. As shown in Fig. 4b, the composite materials contained large amounts of O element, which came from montmorillonite. Whereas, Fe and Ni elements were observed as relatively fewer by EDS mapping observations.

**Figure 5:**
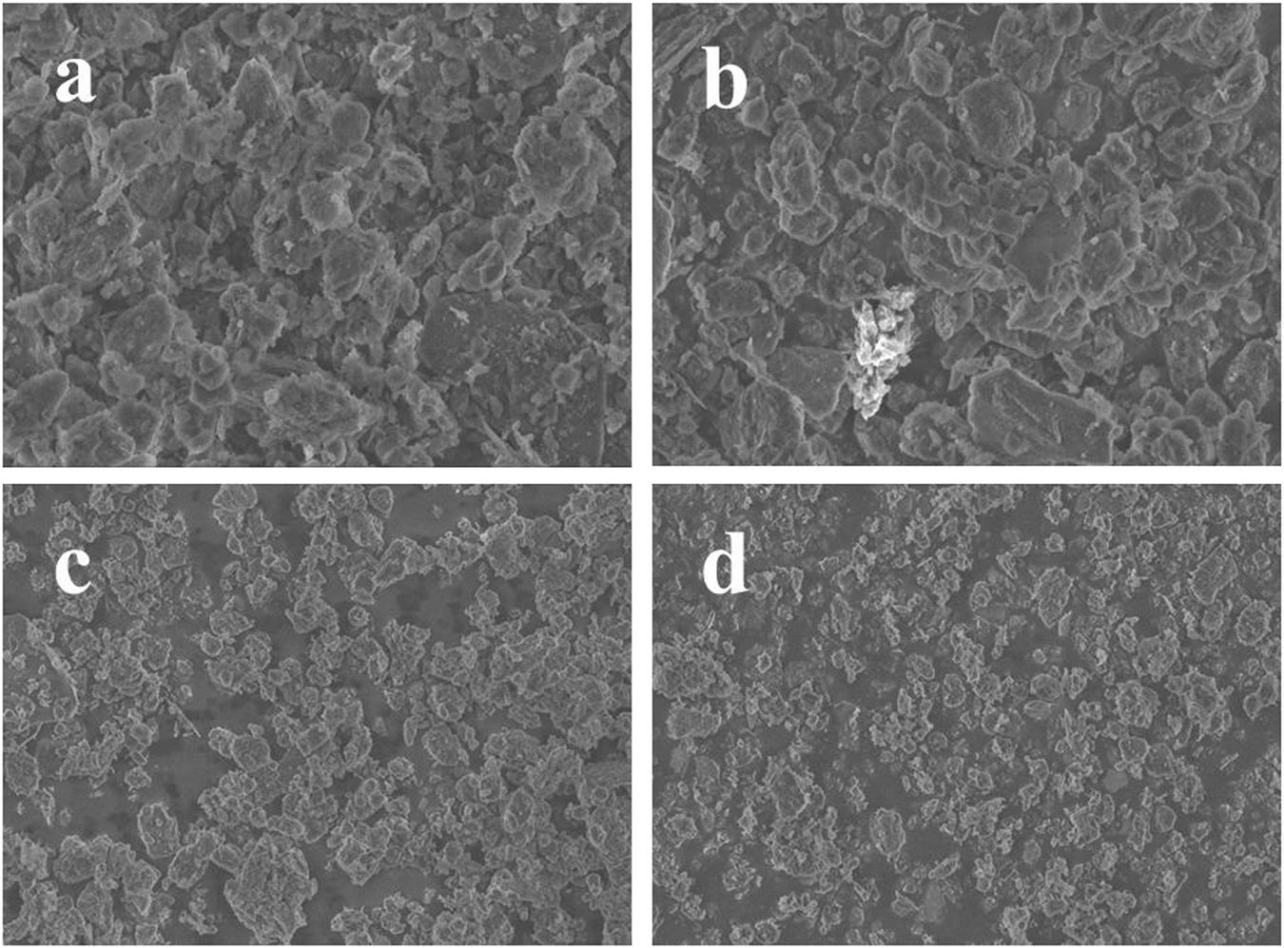
SEM micrographs of Fe/Ni-montmorillonite materials obtained by different reduction temperature: (**a**) 150 °C, (**b**) 250 °C, (**c**) 400 °C, (**d**) 600 °C.

As shown in Fig. 5, with the increase of reduction temperature, the size of the Fe/Ni-montmorillonite materials were smaller, and the degree of dispersion of them were better. Reports mentioned the importance of catalytic materials’ size and uniformity for dechlorination^19^. The smaller the particle size of the material, the larger the specific surface area of the materials. It is generally believed that the larger the surface area of the catalyst, the more active sites on the surface^48^.

**Figure 6:**
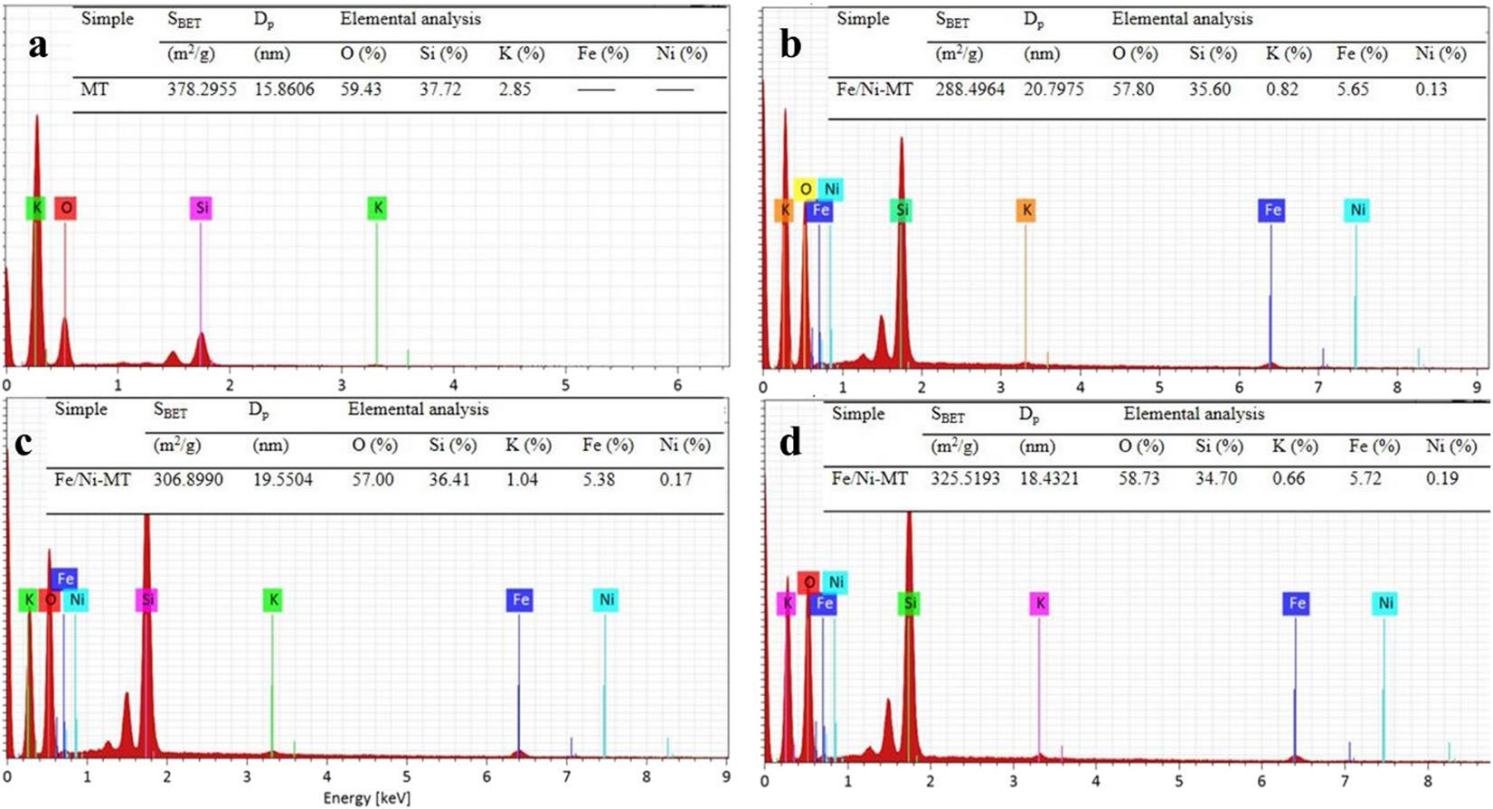
Surface area, average particle size and EDS spectra of Fe/Ni-montmorillonite materials with different Fe/Ni ratio: (**a**) pure montmorillonite, (**b**) 17.7%, (**c**) 28.5%, (**d**) 33.6%.

The surface area and average particle size of Fe/Ni-montmorillonite materials were listed in Fig. 6. The BET surface areas of pure montmorillonite were 378.2955 m^2^/g, while for the materials with 17.7%, 28.5% and 33.6% Fe/Ni ratio, the BET surface areas were 288.4964 m^2^/g, 306.8990 m^2^/g and 325.5193 m^2^/g, respectively. The results indicated that the surface area clearly increased with the growth of Fe and Ni particles on the montmorillonite surface. The average particle sizes of above samples were 15.8606 nm, 20.7975 nm, 19.5504 nm and 18.4321 nm, respectively. The elemental analysis by EDS was showed in Fig. 6. Based on this analysis, montmorillonite consisted of a large amount of O and Si elements. The prepared samples consisted of 0.13%, 0.17%, 0.19% nickel, when the Ni/Fe ratios added in the synthesis process were 17.7%, 28.5%, 33.6%, respectively.

**Figure 7:**
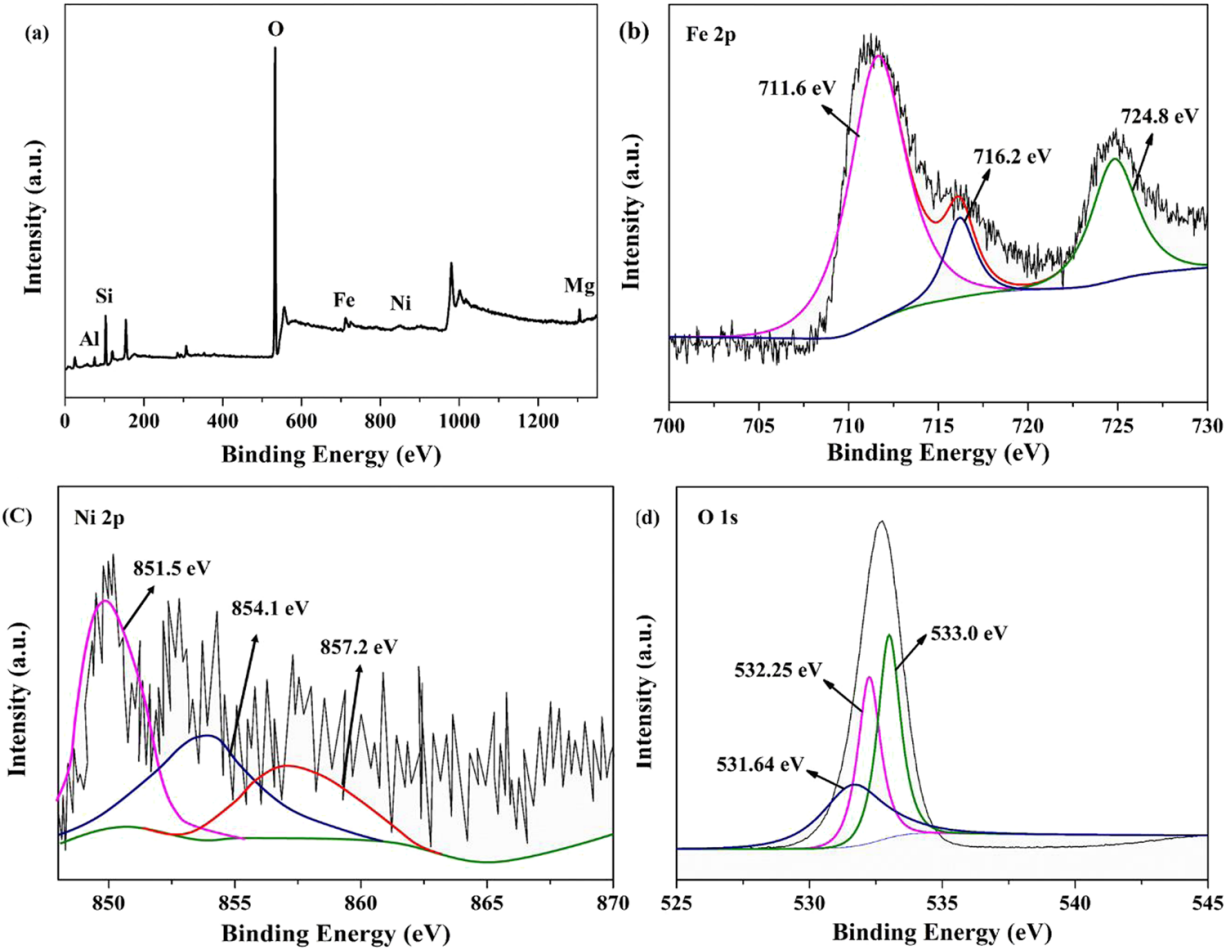
XPS survey scan of (**a**) Fe/Ni montmorillonite, (**b**) XPS Fe 2p spectrum, (**c**) XPS Ni 2p spectrum, (**d**) XPS O 1s spectrum.

Figure 7 showed the XPS result of Fe/Ni-montmorillonite. The full survey XPS spectrum (Fig. 7a) indicated that Fe/Ni-montmorillonite comprised Fe, Ni, O, Si, Al and Mg elements. The Fe spectrum shown in Fig. 7b revealed that the Fe element of Fe/Ni-montmorillointe existed in two valent states, corresponding to Fe° at 711.6 eV, Fe^3+^ at 716.2 eV and 724.8 eV. The Ni spectrum (Fig. 7c) displayed that Ni° at 851.5 eV and Ni^2+^ at 854.1 eV, 857.2 eV. The O spectrum presented in Fig. 7d was best fitted with three components, corresponding to O^2−^ at 531.64 eV, OH^−^ at 532.25 eV, H_2_O at 533.0 eV^49^. The XPS results for Fe/Ni-montmorillonite demonstrated that Fe° and Ni° were successfully synthesized by dry *in-situ* hydrogen reduction.

### Optimization of montmorillonite-templated Fe/Ni bimetal material prepared by dry ***in-situ*** hydrogen reduction

**Figure 8:**
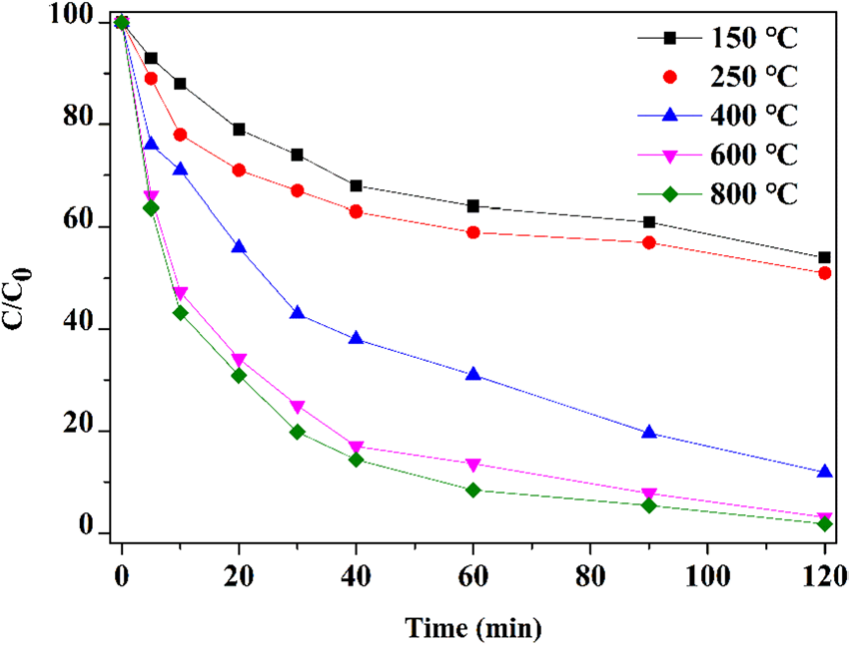
Effect of reduction temperature on the dechlorination efficiency of 4-CP.

The reduction temperature of the preparation process and the content of catalytic metal in bimetal have great influences on the catalytic activity^19^. In general, appropriate amounts of nickel and higher reduction temperature are beneficial to the dechlorination reaction. Figure 8 showed the influence of different reduction temperatures on the preparation of Fe/Ni montmorillonite process towards the dechlorination rate of 4-CP. It could be observed that the reaction rate gradually increased with the increase of reduction temperature. The 4-CP removal was reached almost 100% when the reduction temperature was 800 °C, and Ni content was 28.5%. Studies have shown that low roasting temperature is notably conductive to the improvement of catalytic performance^19^. When the temperature is low, the interaction between the active component and the carrier is weak. During the reduction process, the metals can easily migrate to form large particles^50^. Nevertheless, the 4-CP removal was reached over 90% and a small increase in catalytic activity could be observed with the increase of reduction temperature from 400 °C to 800 °C. Taking into account the cost, 400 °C was chosen as the optimal temperature and used for further experiment.

**Figure 9:**
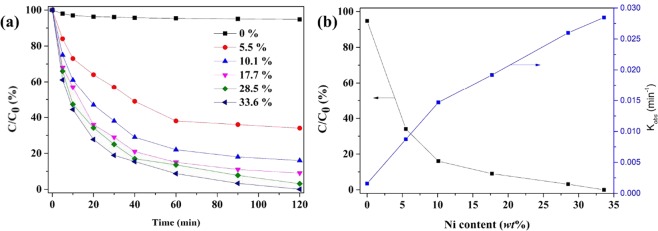
Effect of (**a**) Ni content on dechlorination performanceand (**b**) reaction rate of 4-CP.

Xu *et al*. and Zhang *et al*.^19,48^ reported that Ni could be considered as a better choice of catalyst to dechlorinate 4-CP. Thus, the effect of Ni content (Ni/Fe mass ratio during sample preparation) on 4-CP dechlorination by montmorillonite-template Fe/Ni bimetallic system was investigated. As can be seen in Fig. 9, the dechlorination efficiencies and reaction rates increased with the increase of Ni content. The removal of 4-CP was obtained 100% within 120 min of reaction time when the Ni content reached 33.6%. As shown in Fig. 9a, only a very slight increase in the catalytic activity was observed with the increase of Ni content after 17.7%. Thus, an increase in the catalytic activity with increasing Ni content can be mainly attributed to the presence of Ni on the Fe particles surface, which could stimulate the formation of atomic hydrogen. The results agreed with previous researches^48,51^. This implies that the percentage Ni loading on Fe/Ni nanoparticles might be one of the important parameter to accelerate the reactivity by preventing the formation of iron hydroxide precipitate and releasing the molecular hydrogen^51^. The materials synthesized by this study exhibited excellent removal ability for pollutants in water, and the performance comparisons were shown in Table 1 ^39,41,49,52^.

**Table 1:** Comparison of the performance of Fe/Ni-montmorillonite with other materials.

Samples	Dosage (g/L)	Contaminant	Concentration (mg/L)	Removal (%)	Ref
Fe/Ni-montmorillonite	10	4-CP	20	100	This study
MnO_2_ nanosheets @MMT	10	MB	10	86	^39^
Fe^0^/Ni^0^-montmorillonite	19.17	Biphenyl	1.2	63	^41^
CMC-Fe	0.1	chloroform	10	37	^49^
Attapulgite-Fe/Ni	3.0	BDE47	2.0	62	^52^

## Conclusion

The montmorillonite-templated Fe/Ni bimetallic material prepared by dry *in-situ* hydrogen reduction method exhibited an excellent dechlorination activity for 4-CP. The high dechlorination activity of montmorillonite-templated Fe/Ni bimetal could be attributed to the homogeneous dispersion of Fe and Ni particles on the montmorillonite. Composite materials prepared by this method can efficiently prevent ZVI and Ni from reacting with water and being oxidized. In addition, this method could be easily scaled-up to industrial applications. Montmorillonite played a dominant role in the composite materials because it could inhibit the agglomeration of Fe/Ni particles. The montmorillonite-templated Fe/Ni bimetal materials prepared by dry *in-situ* hydrogen reduction method could be a promising material for the removal of other COCs from aqueous solutions.

## Methods

### Pre-treatment of montmorillonite

5.0 g of montmorillonite was dissolved in 100 mL of deionized water and then stirred for 24 h. The pH of the suspension liquid was adjusted to 6.8 using sodium acetate buffer solution (0.5 M, pH = 5) and added 700 mL deionized water to the solution. pH was maintained for 30 min, then the suspension liquid was separated by centrifugation at 3500 rpm/min for 15 min. The flocculent sediment was dissolved in 400 mL of deionized water and kept in a shaker at 120 rpm for 8 h. The solution was centrifuged at 50–60 g centrifugal force for 6 min, leave the supernatant, and centrifuge the supernatant at high speed. Clay particles (<2 μm) were obtained and stored for later use.

### Montmorillonite-templated Fe/Ni bimetallic prepared by dry ***in-situ*** hydrogen reduction

Initially, 0.1 M FeCl_3_ solution and 0.1 M FeCl_3_-NiCl_2_ mixed solution with different Ni/Fe mass ratio (5.5%, 10.1%, 17.7%, 28.5%, 33.6%) were prepared. 1 g montmorillonite and 30 ml of FeCl_3_ or FeCl_3_-NiCl_2_ solution were mixed and kept on a rotary shaker at a speed of 120 rpm for 8 h to ion exchange (Fe^3+^, Ni^2+^ and K^+^, Na^+^). Then, centrifugation was performed to discard supernatant. After that, the sediment was put into the same solution of 30 mL for ion exchange process. Subsequently, the procedure was repeated for over 6 more times. The material obtained from ion exchange was washed for 5–6 times until there was almost no Cl^−^ in the supernatant. Silver nitrate solution was used to confirm the presence of Cl^−^. The clay powder was collected after drying at 60 °C for 12 h. Finally, the montmorillonite-templated Fe/Ni was prepared by hydrogen reduction in a tube furnace at different temperatures (150 °C 250 °C, 400 °C, 600 °C, 800 °C). The heating rate, hydrogen flow rate and reduction time were set at 20 °C/min, 150 ml/min, and 6 h, respectively.

### Dechlorination experiments

50 ml of 4-CP solution (20 mg/L) was adjusted to an initial pH of 2. Then, 500 mg of montmorillonite composite material was added to the 4-CP solution. The reaction system was kept on a rotary shaker at 220 rpm. The samples were taken at different intervals by an injector with a 0.45 µm nylon membrane.
